# Carotid web stent for the prevention of recurrent stroke: Case report and literature review

**DOI:** 10.1002/ccr3.5473

**Published:** 2022-02-15

**Authors:** Mohammed Alnajjar, Yahia Z. Imam, Naveed Akhtar, Elmukhtar Habas, Ayman Zakaria

**Affiliations:** ^1^ 36977 Division of Internal Medicine Hamad Medical Corporation Doha Qatar; ^2^ Division of Neurology Hamad Medical Corporation Doha Qatar; ^3^ 36977 Division of Neuroradiology Hamad Medical Corporation Doha Qatar

**Keywords:** carotid web, intervention, neurology, stents, stroke

## Abstract

Carotid web has been identified as one of the missed causes of recurrent stroke. The diagnosis and management of such cases impose a challenge to medical practitioners. This etiology should be kept in mind, especially in case of recurrence of stroke in a similar cerebral territory.

## Graphical abstract

A high index of suspicion toward unusual etiologies should be raised in cases of recurrent stroke in same cerebral territory. One of these usually missed causes is carotid web.

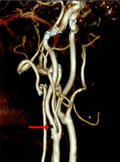

## 1 INTRODUCTION

Large artery atherosclerosis constitutes 16% of total stroke etiologies.^1^ While the prevailing paradigm that the early intervention for severe symptomatic carotid stenosis is beneficial in decreasing recurrence^2^; however, more and more studies have shown that less severe stenosis can cause strokes^3^ and that the size and morphology of the plaques on 3D carotid Doppler can predict recurrences.^4^ A carotid web (CW) is a thin, tissue protrusion from the wall of the carotid artery into the lumen, usually at the origin of the internal carotid artery.

Carotid webs are a rare condition that was initially reported in medical literature over four decades ago.^5^ It is considered to be an underrecognized cause for ischemic stroke particularly among younger patients with a low‐risk factor burden.^5^ There are limited case reports that mention the significance of CW in patients with vascular risk factors. We present a case of recurrent ischemic stroke due to a carotid web that was admitted to Hamad General Hospital (HGH)—Qatar, and he was treated with stenting.

## 2 CASE REPORT

A 64‐year‐old Indian male patient with a past medical history of diabetes mellitus, hypertension, and a recent history of right corona radiata stroke, had left‐sided weakness at the time of his initial presentation. The initial Doppler ultrasound (U/S) showed a small, calcified plaque at the left bulb without causing significant carotid stenosis, and the magnetic resonance angiogram (MRA) on the first visit was reported as right common carotid artery (CCA) and proximal right internal carotid arteries (ICA) atherosclerotic plaques attached to the posterior wall. Workup for ischemic stroke including an echocardiogram and a 48‐hour Holter was unremarkable. The patient was discharged to inpatient stroke rehabilitation on dual antiplatelets (DAPTs) for 2 weeks and subsequently discharged home with total recovery and modified Rankin Score (mRS) of zero.

**FIGURE 1:**
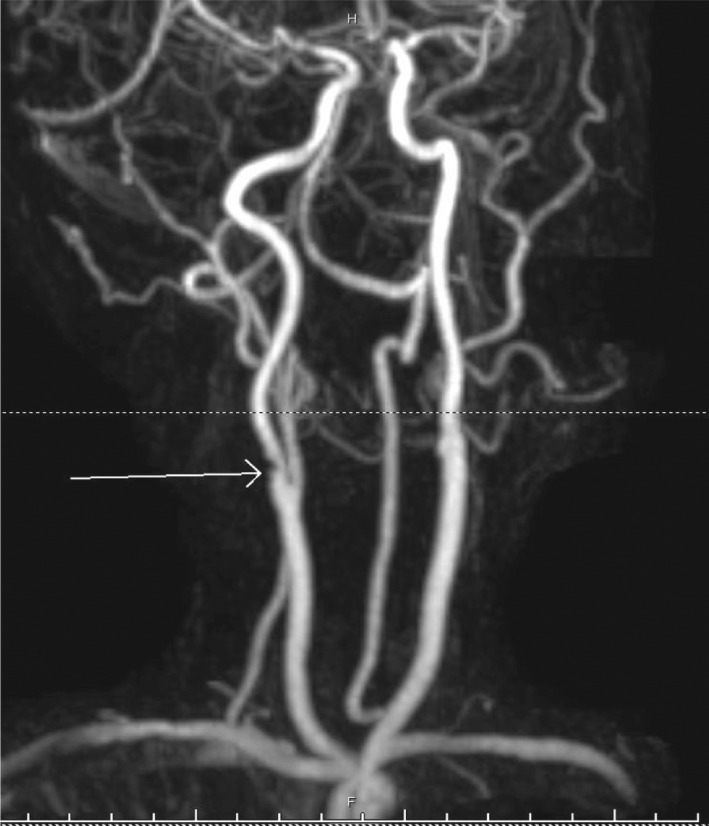
MRA of the neck vessels showing a shelf‐like projection in the posterior wall of the ICA just distal to the bifurcation

**FIGURE 2:**
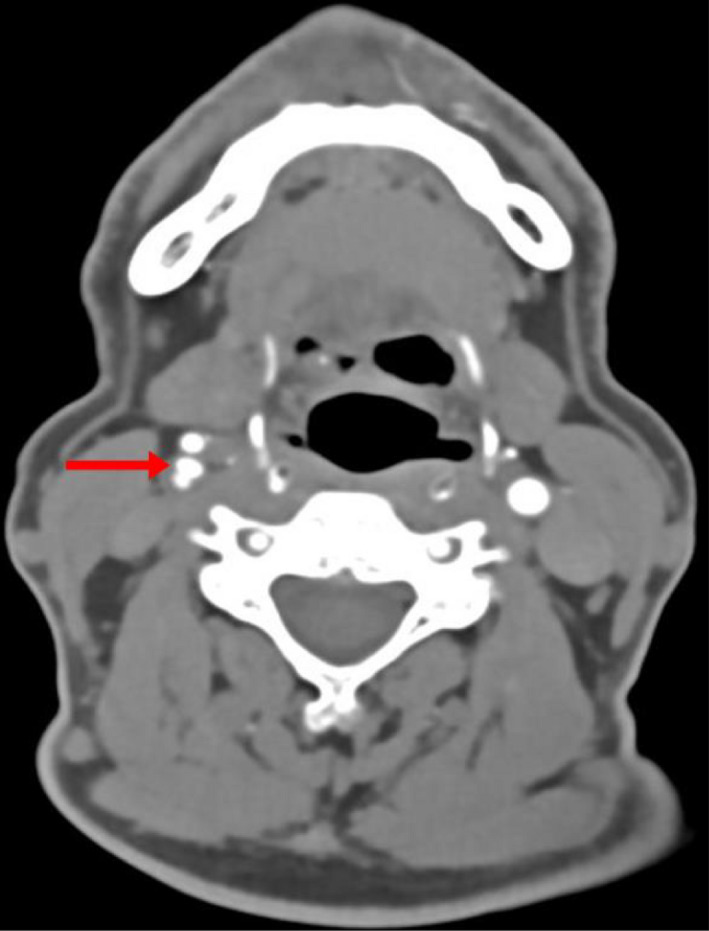
CTA of the neck, axial sections showing a triple lumen sign just distal to the right common carotid artery bifurcation. The normal external carotid artery (ECA) anteriorly and the ICA posteriorly divided into two separate lumens with fine line

**FIGURE 3:**
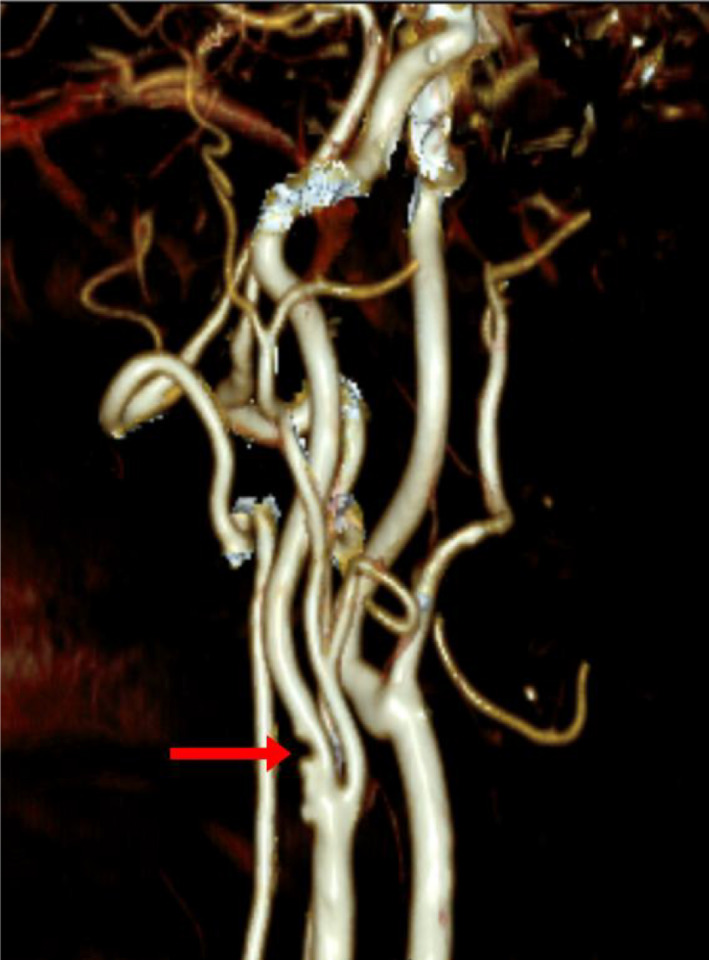
3D reconstructed CTA showing irregularity of the posterior wall of proximal right ICA

Six weeks after the first visit the patient was readmitted with left‐hand weakness, that occurred suddenly, with no associated symptoms. Magnetic resonance imaging (MRI) head was repeated and showed new foci of infarction in the right junction of both territories of anterior and posterior cerebral arteries. Doppler U/S of the carotids was repeated, which did not show any change in comparison to the previous U/S. MRA was also repeated and was discussed with stroke neurology and neuroradiology, and suspicion of right ICA web was raised given the discrepancy between the Doppler and the MRA (Figure 1). This was confirmed via computed tomography angiography (CTA) (Figures 2, 3), which showed a 7‐mm hook‐shaped filling defect 4‐mm distal to the carotid bifurcation, causing a 50% stenosis in the right ICA.

**FIGURE 4:**
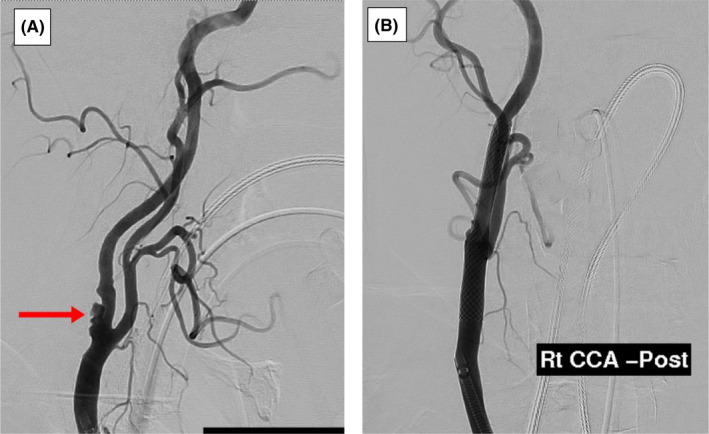
Carotid angiography pre‐ (A) and post‐stenting (B): There are filling defects on the posterior wall of the right CCA & ICA in (A) corrected smooth outline post‐stenting in (B)

Both carotid endarterectomy (CEA) and stenting were offered to the patient, and the patient opted for stenting of the right ICA (Figure 4) and was discharged on DAPT with an almost complete recovery of his left‐hand weakness.

Patient has been followed in stroke clinic till day of publishing this paper, which is seven months after discharge. During this period, he had no recurrence of stroke.

## 3 DISCUSSION

Carotid web is frequently seen in cryptogenic strokes, occurring in young patients with little or no risk factors.^6^ This case is interesting as it shed light on a patient with recurrent stroke with vascular risk factors for atherosclerosis and the development of carotid plaques. Additionally, it exposed the discrepancy between different imaging modalities.

### 3.1 Imaging modalities to diagnose CW

Computed tomography angiogram (CTA) is considered the modality of choice for the diagnosis of carotid web.^7^ Madaelil et al^8^ investigated the strength of agreement between CTA and digital subtraction angiography (DSA), and between CTA and carotid ultrasound (US). The results showed that there was a significantly better correlation between CTA‐DSA than CTA‐US (Z = 3.58; *p* = 0.0003). No clinical trial has been done to compare CTA with MRA.

In Zhu et al, eight carotid web cases have been identified retrospectively. Strangely, CTAs of three cases were reported negative initially. However, US was able to detect carotid web in these cases. U/S was also shown to have superiority in detecting thrombus on top of the web.^9^

Evidence regarding MRA for carotid web detection is very scarce, there is a case series and two case reports that investigated the MRI findings of carotid webs. In Boesen et al,^10^ the carotid webs were reported retrospectively, and the detection rate was 100% of the five reported cases, with 4 of them showed thin septum protruded just proximal to the carotid bifurcation, while the position was at the level of bifurcation in one case.

In the case report they used T1W and TOF images, the former showed a crescentic hyperintense lesion. Flow abnormality suggestive of turbulent flow pattern was seen on 2D TOF images.^11^ Another case reported the finding of hyperintense film‐like lesion on T2 images.^12^

### 3.2 Management of CW

As per literature, the proposed treatment modalities for such cases are either by CEA, stenting, or medical treatment with antiplatelets.

Aggressive therapy is required if a CW is detected ipsilateral to an acute stroke given the high rate of stroke recurrence while on antiplatelet therapy.^13^ In a prospective study done on cases of cryptogenic stroke, 32% of the cases had a recurrent stroke in the same area, 9% happened while patients were on DAPT.^14^

Joux et al,^15^ followed up 25 patients prospectively over 5 years, 30% of medically treated patients developed a recurrent stroke in the same territory. The median time for recurrence was 12 months, with the earliest observed at 1 month. It is worthwhile to note that this study did not mention whether single or dual antiplatelets were used.

Anticoagulation is attractive academically with reports of clots uncovered from the web akin to the left atrial appendage; however, there are scarce clinical data to recommend its habitual use.^16^

Both CEA and stenting have been used for CW treatment. While CEA appears to be a more definite treatment in terms of reducing recurrence, it poses a higher upfront risk of complication, whereas stenting is less invasive, with less upfront risk. However, it is sometimes associated with a long‐term risk of recurrence.^17^, ^18^

## 4 CONCLUSION

CW should be kept in mind as a cause of recurrent stroke, even in patients with multiple risk factors for cerebrovascular accidents, especially if recurrence happened in the same territory.

Moreover, the presence of a discrepancy between U/S and CTA/MRA should increase the index of suspicion for CW as a culprit for recurrence. Till now, the literature review showed that CTA is superior to US for CW diagnosis, and aggressive intervention is warranted in such cases as the recurrence rate even with DAPT is unacceptably high.

## CONFLICT OF INTEREST

This manuscript has not been submitted for publication elsewhere. All authors have reviewed and approved the manuscript before submission. None of the authors have any conflict of interest to declare.

## AUTHOR CONTRIBUTIONS

Mohammed Alnajjar involved in manuscript writing, literature review, review, and approval of the final manuscript. Yahia Z Imam involved in manuscript writing, literature review, revisions in manuscript, review, and approval of the final manuscript. Ayman Zakaria involved in case identification, radiological imaging interpretation, literature review, and approval of the final manuscript. Elmukhtar Habas involved in manuscript revisions, literature review, critical review, and approval of the final manuscript. Naveed Akhtar involved in case identification, manuscript revisions, literature review, critical review, and approval of the final manuscript.

## ETHICAL APPROVAL

Ethics approval was taken from Medical Research Center (MRC) Qatar before submission of this manuscript.

## CONSENT

Written informed consent was obtained from the patient for publication of this case report and accompanying images. A copy of the written consent is available for review by the Editor‐in‐Chief of this journal on request.

## Data Availability

Data sharing is not applicable to this article as no datasets were generated or analyzed during the current study.
